# Recurrence of progressive multifocal leukoencephalopathy despite immune recovery in two HIV seropositive individuals

**DOI:** 10.1007/s13365-015-0419-y

**Published:** 2016-01-04

**Authors:** Kate M. Crossley, Shruti Agnihotri, Joga Chaganti, Michael L. Rodriguez, Leon Patrick McNally, Nagagopal Venna, Sarah E. Turbett, Matthew Gutman, Adrienne Morey, Igor J. Koralnik, Bruce J. Brew

**Affiliations:** Department of Neurology, St Vincent’s Hospital, Level 4 Xavier Building, Sydney, 2010 Australia; Division of Neuro Immunology, Center for Virology and Vaccine Research, Beth Deaconess Medical Center, Boston, USA; Department of Radiology, St Vincent’s Hospital, Sydney, Australia; Department of Neuropathology, St Vincent’s Hospital, Sydney, Australia; Department of Molecular Medicine, St Vincent’s Hospital, Sydney, Australia; Department of Neurology, Massachusetts General Hospital, Boston, USA; Department of Infectious Diseases, Massachusetts General Hospital, Boston, USA; Department of Neurosurgery, St Vincent’s Hospital, Sydney, Australia; Department of Anatomical Pathology, St Vincent’s Hospital, Sydney, Australia; Department of Neurology, St Vincent’s Hospital, Sydney Australia, also Peter Duncan Neurosciences Unit Neurosciences Program, St Vincent’s Centre for Applied Medical Research, Sydney, Australia; Department of Infectious Diseases and Immunology, St Vincent’s Hospital, Sydney Australia, also Peter Duncan Neurosciences Unit Neurosciences Program, St Vincent’s Centre for Applied Medical Research, Sydney, Australia

**Keywords:** Progressive multifocal leukoencephalopathy, Neuroimmunology, Neurovirology

## Abstract

We present two cases of recurrent progressive multifocal leukoencephalopathy (PML) in patients with long standing virally suppressed human immunodeficiency virus (HIV) and normal CD4+ T cell count who were taking stable regimens of highly active antiretroviral therapy (HAART). This has significant implications for other patients with a past history of PML, not just those with HIV but also those on medications such as natalizumab or fumarates.

## Introduction

Progressive multifocal leukoencephalopathy (PML) usually affects immunocompromised people, such as those with advanced HIV or a haematological malignancy. A minority of initial PML diagnoses are in people who are minimally or mildly immunosuppressed (Berger et al. 1998; Gheuens et al. 2010). Management is to restore immune function where possible, and this can result in remission of the PML. The two cases we describe had initial remission of PML, with subsequent development of symptoms and investigation findings consistent with recurrent PML despite interval maintenance of minimal immunosuppression and HIV suppression. This has not previously been described.

## Case one

A 49 year old man presented with chronic headache in 2013, on a background of HIV diagnosed in 1987.

In the early 2000s his CD4+ T cell count was at a lifetime nadir of 70 cells/μl, plasma HIV RNA 5 · 35 log, and cerebrospinal fluid (CSF) 4 · 69 log. At that time he had cognitive impairment consistent with HIV associated neurocognitive disorder (HAND) with an MRI brain showing multifocal grey and white matter hyperintensities. CSF was normal including negative PCR for JC virus (JCV) DNA, but a right frontal lobe biopsy showed oligodendrocyte nuclei with viral inclusions, prominent reactive astrocytes including bizarre forms, and was strongly positive for JCV DNA by PCR. HIV associated encephalitis was also present (Anders et al. 1986).

He commenced HAART for the first time with efavirenz, lopinavir/ritonavir, lamivudine, and stavudine (the latter two later switched to raltegravir for tolerability), with consequent CD4+ T cell count rise to over 300 cells/μl. He was clinically stable but required institutional care due to poor cognition that did not improve with HAART.

**Fig. 1:**
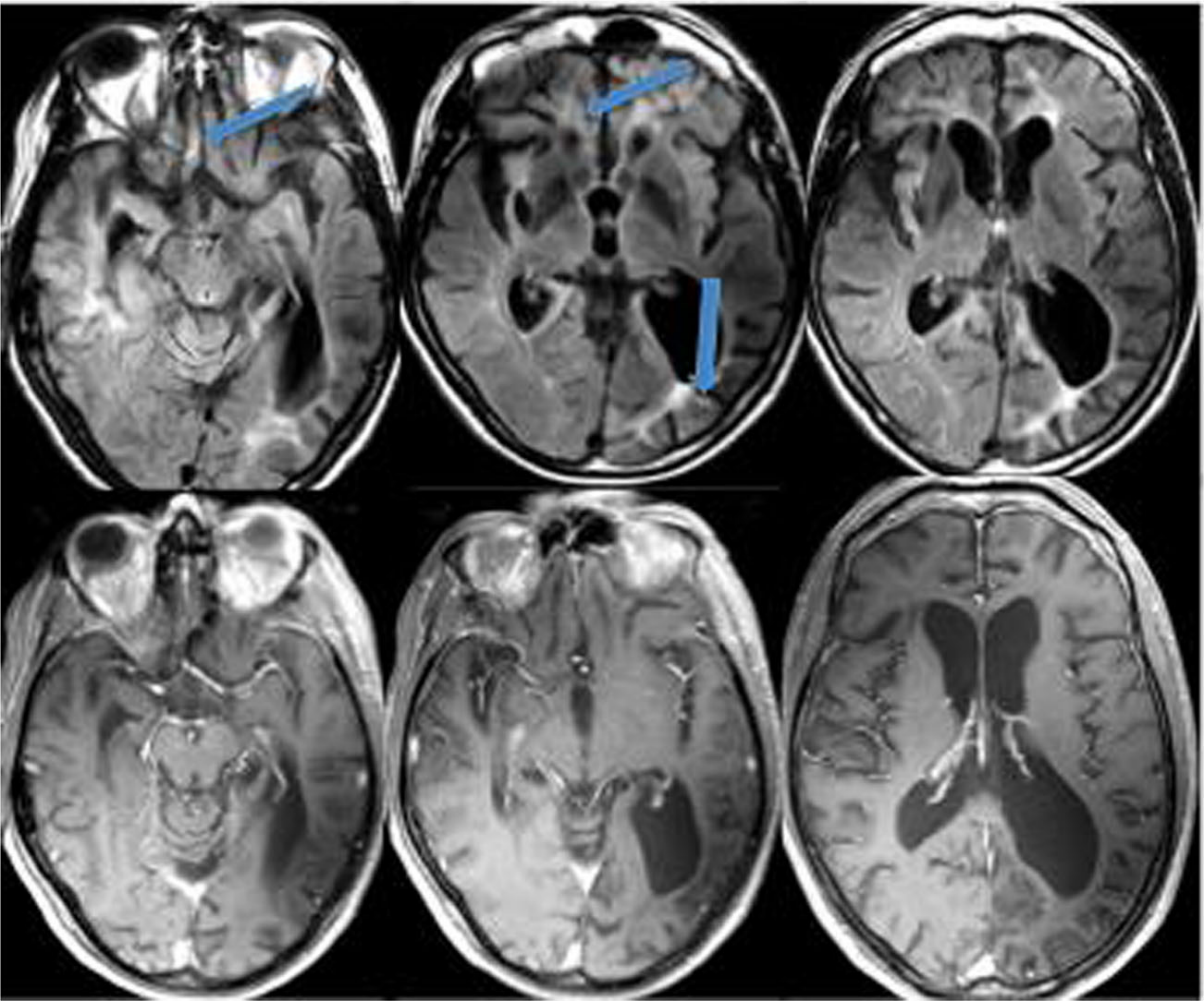
Case one, 2008: Fluid attenuated inversion recovery (FLAIR) MRI sequences showing multifocal hyperintensity in the subcortical U-fibres of the right basifrontal, right temporal, and left parieto-occipital regions (see *arrows*). The corresponding T1 post contrast images (*lower row*), demonstrate an absence of enhancement

**Fig. 2:**
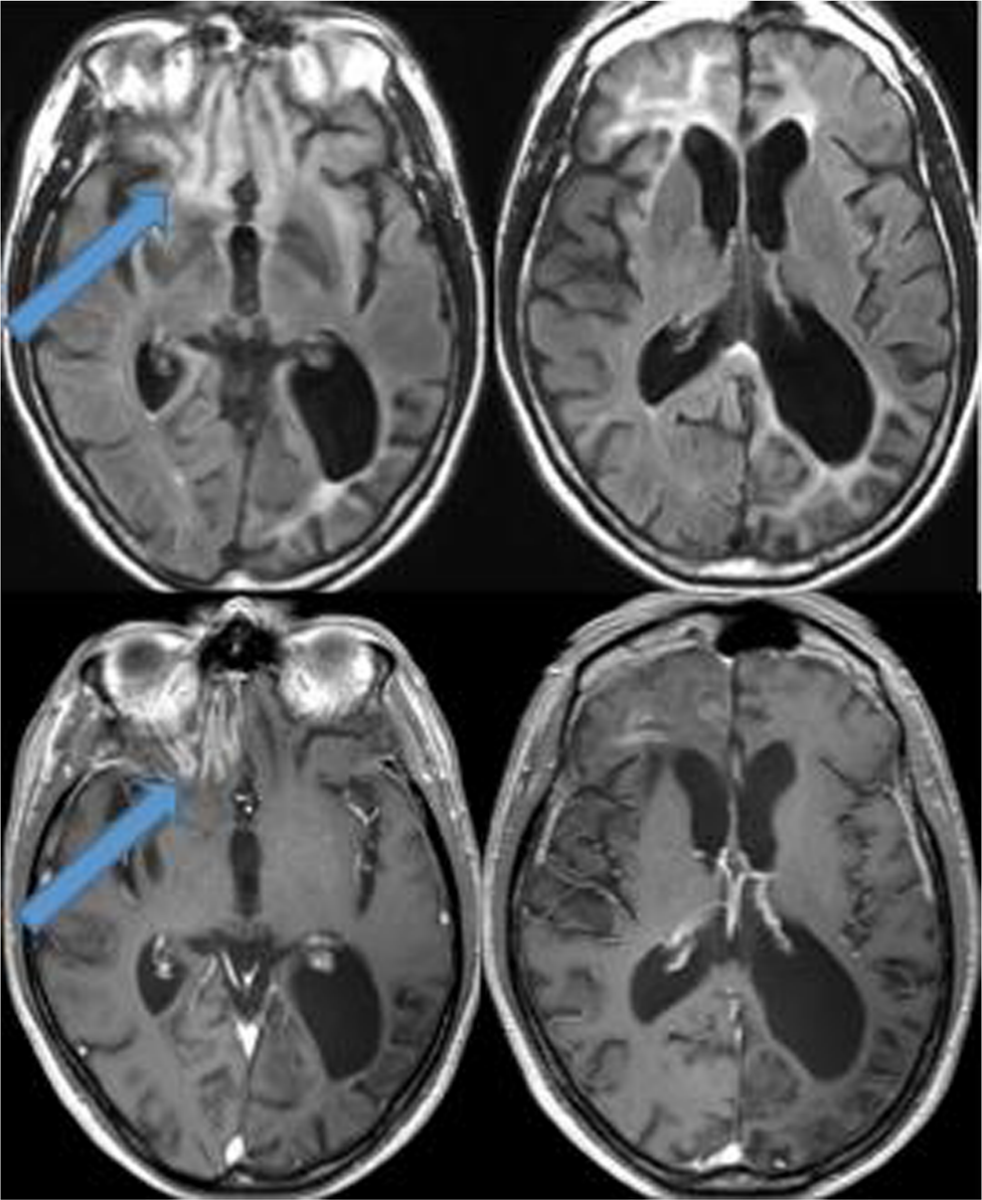
Case one, 2011: FLAIR MRI sequences from 2011 again show hyperintensity in the right basifrontal (see *arrows*) and left parieto-occipital regions, now with enhancement on corresponding post contrast T1 images (*lower row*) in the right basifrontal region only

**Fig. 3:**
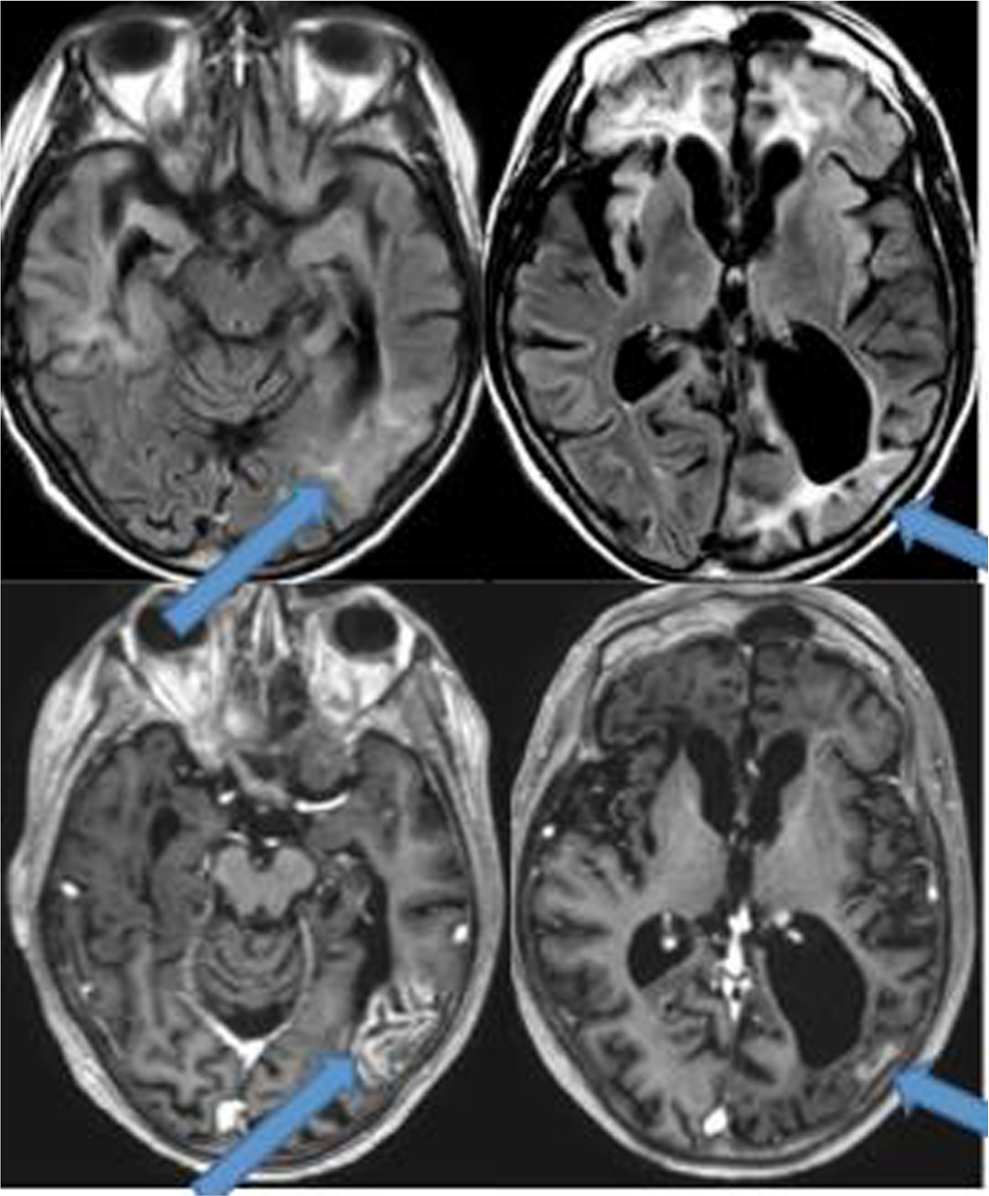
Case one, 2013: FLAIR hyperintensity has increased in the left parieto-occipital region (see *arrows*, *top images*), with new gyriform enhancement (see *arrows*, *bottom images*) on corresponding pre-contrast FLAIR sequences. The right basifrontal contrast enhancement has shown a complete resolution

By 2013 his CD4+ T cell count had been over 500 cells/μl for 7 years, and HIV RNA had been undetectable for a decade. Annual MRI brain scans since 2008 (Figs. 1, 2 and 3) showed progressive white matter atrophy, and since 2010 asymptomatic fluctuating contrast enhancement without mass effect, in both cortical grey and subcortical white matter.

Examination when he presented with headache in 2013 revealed a new right inferior quadrantanopia, preexisting cognitive impairment, mild upper limb action tremor, and impaired tandem gait. CD4+ T cell count was 648 cells/μl. CSF was bland; cryptococcal antigen and HIV RNA were undetectable. However CSF JCV DNA was detected (below 30 copies/mL, the lower limit of quantification of the assay), using primers targeting the VP2 gene. The sequenced 131 base pair PCR product was a 93 % match to JCV CPN1 strain using the Basic Local Alignment Search Tool. MRI brain showed increased subcortical signal hyperintensity and cortical enhancement in the left posterior temporal lobe extending into the parieto-occipital region (Fig. 3). He was diagnosed with possible recurrent PML.

His headache spontaneously improved only to recur several months later. An MRI showed new enhancing lesions in the left temporoparietal grey and subcortical white matter. Serial MR spectroscopic studies showed increasing choline and myoinositol in these regions with markedly reduced N-acetylaspartate implying ongoing gliosis of the subcortical U-fibres. CSF again showed low JC viral load. A stereotactic-guided biopsy of the left temporoparietal lesion was performed within a month of presentation, and repeated six months later because of the unusual findings, with essentially the same result. The patient has shown no clinical progression on history or examination since the biopsies. MRI has not been repeated.

## Pathological findings

The leptomeninges were fibrotic with increased vascularity and patchy chronic inflammation, predominantly T cells. The cortex displayed prominent reactive gliosis, microglial activation with multifocal microvascular proliferation, mild neuronal loss, demyelination with partial axon preservation, sparse T cells (predominantly CD8+), and some haemosiderin deposition. There were scattered dysmorphic neurons, some with enlarged nuclei, marginated chromatin, and peripherally displaced Nissl substance, and several bizarre astrocytes with enlarged hyperchromatic pleomorphic nuclei.

There were frequent polyomavirus immunoreactive nuclei mostly in astrocytes and occasional neurons (SV40 large T antigen Ab cross-reacting with JCV). Most of the positive nuclei were not within the areas of demyelination or microvascular proliferation. Classic oligodendroglial inclusions and microglial nodules, noted in the first biopsy, were not identified. Immunostains for HIV p24, CMV, Toxoplasma, and HHV8 were negative.

## Case two

A 54-year-old female reported new gait difficulties and unsteadiness in 2012, with associated recent decline in memory and language.

**Fig. 4:**
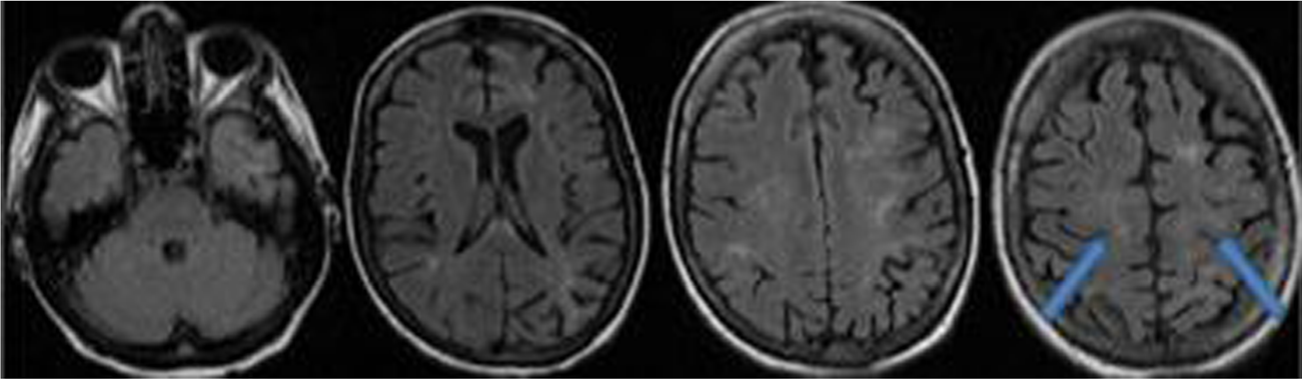
Case two, 2006: FLAIR MRI sequences demonstrating multiple poorly defined bright foci in the central and periventricular white matter, with subcortical U-fibre involvement (see *arrows*) in the frontoparietal region

In 2006 HIV was diagnosed after presenting with memory impairment. At the time her nadir CD4+ T cell count was 186 cells/μl with an HIV RNA viral load of 5 · 28 log. An MRI showed left frontotemporal white matter lesions (Fig. 4). CSF was qualitatively positive for JCV DNA by PCR. No quantitative test was performed. She improved significantly on a stable regimen of tenofovir, emtricitabine, and efavirenz. Her HIV RNA was below detection on all subsequent testing.

**Fig. 5:**
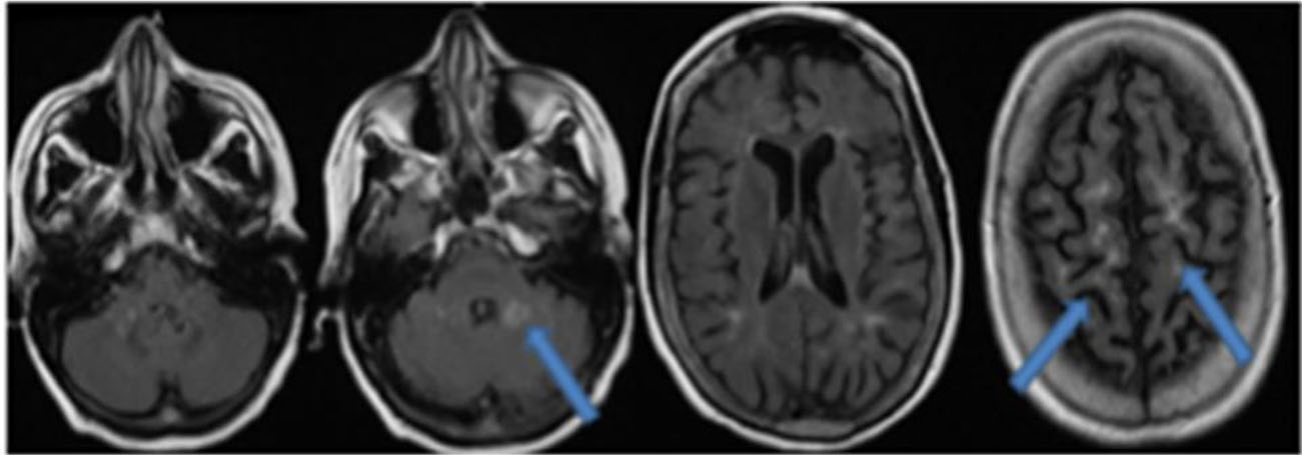
Case two, 2012: FLAIR MRI sequences showing new lesions in the posterior fossa (see *arrow*) and an increase in the number of lesions in the high frontoparietal white matter bilaterally (see *arrows*). There was also increased cerebral atrophy. There was no mass effect, perilesional oedema, or enhancement after contrast administration (not shown)

MRI in 2012 showed significant atrophy (predominantly left frontoparietal) and new non-enhancing T2 hyperintensities in the middle cerebellar peduncles, splenium and periventricular region (Fig. 5), mainly involving white matter, but also grey-white matter junction.

CSF was positive for JCV DNA at 2786 copies/ml and negative for HIV RNA. She had a detectable immune response to JCV mediated by both CD4+ and CD8+ T cells. Although her CD4+ T cell count was 676 cells/μl and HIV RNA undetectable, her medications were changed to abacavir, emtricitabine, etravirine, raltegravir, and maraviroc to optimise brain penetration. The right frontal region was biopsied. Mirtazapine was trialled to no avail, and she deteriorated and died seven months after the biopsy.

## Pathological findings

The biopsy showed marked reactive white matter gliosis, and acute demyelination. There were scattered large, atypical nuclei, some suggestive of viral inclusions. Immunostaining using SV40 Ab highlighted scattered positive cells, mostly oligodendrocytes in the white matter edge of the demyelinating lesions, and the grey-white junction.

HIV encephalitis was present with marked microglial activation (highlighted with CD45 immunostaining) occasional microglial nodules and reactive gliosis with mild neuronal loss (highlighted with NeuN). Additionally there was mild chronic inflammation, with perivascular and scattered parenchymal CD4+, CD8+, and CD3+ T cells which could not be accurately quantified because of the limited tissue available for study.

## Discussion

These cases demonstrate three novel important points. First, PML may recur years after the initial illness, despite normal or near-normal immune function. Second, recurrence of clinical PML may be presaged by MRI brain changes. Third, the MRI changes may fluctuate over time.

We believe JCV caused the clinical, radiological, and neuropathological features described. In both cases, clinical and MRI findings were compatible with PML (Brew et al. 2010; Gheuens et al. 2013), although in the first case, the fluctuating cortical gyriform enhancement is unusual. The pathology was also consistent with PML except the microvascular proliferation. Hyperperfusion within and at the edge of PML lesions has previously been demonstrated, however it was unclear whether this was due to microvascular proliferation or vasodilation (Khoury et al. 2013).

SV40 staining was seen in cortical neurons in the first case. This has been described in up to half of patients with classic PML, within areas of cortical demyelination or in isolation (Wuthrich and Koralnik 2012). Our patient’s biopsy showed cortical demyelination with infection of astrocytes and neurons and some inflammation. This is consistent with cortical involvement by PML, which may explain the cortical gyriform enhancement seen on MRI.

Most importantly, JCV DNA was detected in the CSF and brain of both patients by several techniques. No alternative cause was found. Autopsies of HIV infected patients have previously shown “burnt out” cerebral lesions from long term PML without evidence of JCV activity (Gray et al. 2003). There is no clear reason why these two patients in particular had PML recurrence; hypotheses include an undetermined host genetic predisposition, HIV-accelerated age related immune senescence, or viral factors such as development of a mutation in a JCV quasi-species.

JCV DNA has previously been found in low copy number in CSF up to one half of natalizumab associated PML patients months (and in one case three years) after PML stabilization off natalizumab (Ryschkewitsch et al. 2010). However those patients had no definite evidence of clinically active disease in contradistinction to our two patients.

These cases therefore have significant implications for the prognosis and management of patients with JCV CNS infection. Although PML often improves with HAART, our findings suggest this population, and others with PML, may be at risk of PML recurrence. Serial monitoring clinically and by MRI may allow early identification. There is no proven PML therapy besides immune reconstitution (Tan et al. 2009; Koralnik 2006), which is of particular concern given that our patients were not immunosuppressed anymore. However, should PML recurrence be identified in other groups such as natalizumab or fumarates-treated patients, current immunomodulating and immunosuppressive therapies may need to be reconsidered.
